# Aquaporin-4 Water Channel in the Brain and Its Implication for Health and Disease

**DOI:** 10.3390/cells8020090

**Published:** 2019-01-27

**Authors:** Simone Mader, Lior Brimberg

**Affiliations:** 1 Institute of Clinical Neuroimmunology, Biomedical Center and University Hospital, Ludwig-Maximilians University Munich, D-82152 Martinsried, Germany; 2 The Feinstein Institute for Medical Research, The Center for Autoimmune, Musculoskeletal and Hematopoietic Diseases, Northwell Health System, Manhasset, NY 11030, USA

**Keywords:** aquaporin-4, autoantibodies, disease, development, neuromyelitis optica spectrum disorder, glymphatic system

## Abstract

Aquaporin-4 (AQP4) is a water channel expressed on astrocytic endfeet in the brain. The role of AQP4 has been studied in health and in a range of pathological conditions. Interest in AQP4 has increased since it was discovered to be the target antigen in the inflammatory autoimmune disease neuromyelitis optica spectrum disorder (NMOSD). Emerging data suggest that AQP4 may also be implicated in the glymphatic system and may be involved in the clearance of beta-amyloid in Alzheimer’s disease (AD). In this review, we will describe the role of AQP4 in the adult and developing brain as well as its implication for disease.

## 1. Introduction

AQP4 belongs to a family of channels that is selectively permeable to water. AQP 1, 4 and 9 are expressed in the mammalian brain [1]. Aquaporin-4 (AQP4) is the most abundant water channel in the brain, spinal cord and optic nerve and controls brain water homeostasis [2,3]. This bidirectional water channel was first described by Agre’s and Verkman´s groups [4,5] who previously named it mercury-insensitive water channel (MIWC) because it could not be inhibited by adding mercury-containing compounds [4]. AQP4 is most abundant in astrocytes and ependymal cells lining in the ventricles with the highest expression on perivascular astrocytes end feet that surround blood vessels in the central nervous system (CNS). Density of AQP4 is greatest on the region of the astrocyte closest to the vessel (also known as polarized expression of AQP4) [2,3]. Loss of AQP4 polarity refers to AQP4 expression being mislocalized and broadly distributed in the astrocyte, rather than being focused on the endfeet surrounding blood vessels [6,7]. Due to its particularly high expression at the blood brain barrier (BBB) and blood cerebrospinal fluid (CSF) barrier, AQP4 controls bidirectional fluid exchange [8].

A growing numbers of neurological conditions are now associated with an alteration in AQP4 expression or localization. An imbalance in water homeostasis in the brain has been associated with pathological conditions such as traumatic brain injury and stroke [9,10]. Increasing evidence suggests that AQP4 is also involved in brain inflammation, glymphatic fluid clearance, synaptic plasticity and memory formation, regulation of extracellular space (ECS) volume and potassium homeostasis [8,11,12]. The involvement of AQP4 in several pathogenic conditions is mainly based on findings in post mortem brain tissue, in vitro studies and the usage of AQP4 deficient rodent models.

A loss of AQP4 polarization in perivascular astrocytic endfeet such as occurs in many brain injuries, may result in BBB breakdown. This may be particular relevant for the aging brain and Alzheimer’s disease (AD) [13]. 

In contrast to the role of AQP4 in the adult brain, little is known about the role of AQP4 during early development in the fetal brain. 

In this review, we will discuss the role of AQP4 in health and will share some novel insights from pathological conditions involving AQP4. 

## 2. AQP4 and Its Role during Development

There are scant data about the role of AQP4 during development. Since AQP4 is expressed in the adult brain on astrocyte endfeet, AQP4 expression during development is mostly considered to be linked to the time astrocytes appear in the brain. In the early postnatal phase of development, astrocytes have been described to contribute to postnatal angiogenesis and the formation of the BBB [14]. Transcriptional analysis of the fetal mouse brain (embryonic day E14.5) showed AQP4 expression in proliferating progenitor cells, much less in differentiated progenitor cells, and none in neurons [15]. Early expression of AQP4 was further supported by a study showing that AQP4 is expressed on radial glia cells in the developing mouse brain [16]. Using immunohistochemistry, AQP4 expression was detected as early as embryonic day E16, yet not in a polarized expression pattern [16]. A functional role of embryonic AQP4 has not been studied so far. One study reported the unexpected occurrence of sporadic obstructive hydrocephalus in a small subset of AQP4 deficient mice [17]. Histological analysis of those offspring revealed aqueductal stenosis, which blocks the CSF flow in the ventricular system, as well as ependymal disorganization. This study suggests a possible involvement of AQP4 in the pathogenesis of aqueduct stenosis, but does not determine if this occurs during neurodevelopment or occurs only later on in aged mice.

## 3. AQP4 and Its Role in the Adult Brain

In the CNS, AQP4 is highly expressed in the perivascular astrocyte foot processes and glial limiting membrane and at lower levels in perisynaptic astrocytic processes [2,3,18]. Thus, AQP4 is expressed at very dense levels at the BBB, however it is also expressed at areas of the CNS that lack a BBB, such as the circumventricular organs [2,3]. 

AQP4 monomers are ~30 kDa [19]. Each monomer consist of 6 transmembrane helices and 2 helices that do not span the membrane entirely [18]. Each monomer has a central aqueous pore. AQP4 is expressed as a tetramer in the plasma membrane [20]. AQP4 exists as 2 major isoforms, M1 and M23 which differ in their translation start sites from methionine M1 (323 amino acids) or methionine M23 (301 amino acids) [21]. Tetramers comprised of M23 form higher order structures, called orthogonal arrays of particles (OAP), which are crystal-like supramolecular assemblies in the plasma membrane [18,22,23]. OAP may consist of co-expressed M1-M23 AQP4 heterotetramers [24]. It was suggested that OAP might enhance water permeability, alter cell-cell adhesion and facilitate AQP4 polarization to astrocyte endfeet [25,26,27], yet the biological significance remains unknown [28]. OAP is the target of anti-AQP4 antibodies in neuromyelitis optica spectrum disorder (NMOSD), in which antibodies target specifically the OAP rather than monomeric M1 AQP4 [29,30,31,32] (see further discussion below).

In addition to the relevance of OAP, little is known about the regulation of the polarized expression of AQP4 at the perivascular endfeet. It has been hypothesized that AQP4 interacts with α-syntrophin or agrin, which both show polarized expression in astrocyte endfoot processes [33]. Contact by astrocytes with endothelial and pial cells may also influence AQP4 polarization, as loss of these contacts in pathological conditions may disturb AQP4 polarization [34,35].

### Insights from AQP4 Deficient Mice

Constitutive AQP4 deficient mice as well as glial-conditional AQP4 deficient mice have been used to understand the role of AQP4 in the brain [7,36].

AQP4 deficient mice revealed a role for AQP4 in cognition and memory. These mice show cognitive deficit in the object location memory test [37] and a spatial memory impairment in the Morris water maze [38]. 

One study suggested that AQP4 deficient mice have an impaired BBB, decreased tight junction proteins and swollen astrocyte endfeet [39], yet, other studies could not replicate these findings and observed no alterations in the BBB [36,40]. 

AQP4 deficient mice show a more pronounced phenotype under certain insults or injury. When the role of AQP4 in brain water balance was studied in non-stressed conditions, no obvious change in water homeostasis was observed in vivo [35]. In contrast, AQP4 deficient mice exposed to stress conditions often show either worse outcomes or beneficial effects depending on the disease model. AQP4 deficient mice have a better survival in an acute water intoxication model for brain edema [41], since in this model AQP4 is facilitating water movement into the brain across an intact BBB. In contrast, AQP4 deficient mice with vasogenic edema have a worse outcome with increased brain swelling, as in this pathological condition, AQP4 is required for edema resolution [42].

## 4. AQP4 during Disease

### 4.1. Role of AQP4 in Inflammation

#### Autoantibodies to Astrocytic AQP4 in NMOSD and Its Disease Spectrum

Over recent years, interest in AQP4 has increased due to the discovery that it is the target antigen in the neurological autoimmune disease Neuromyelitis spectrum disorder (NMOSD) [43,44]. NMOSD is an autoimmune astrocytophathy commonly characterized by astrocyte loss, demyelination in the spinal cord, optic nerve and brain. Typically patients present with demyelinating lesions spanning three or more vertebral segments. Around 80% of NMOSD patients harbor autoantibodies that target AQP4 (AQP4-IgG) in the brain. These antibodies were first discovered upon incubation of serum samples with rodent tissue which resulted in a perivascular binding pattern [43]. Because antibodies to AQP4 are not found in serum of healthy controls and multiple sclerosis (MS) patients [29,45], their presence is a diagnostic criterion for NMOSD [46,47], and allows early diagnosis as the clinical presentation of NMOSD patients can resemble MS and other neurological disease. Since the course of treatment for NMOSD patients is different than from MS, early correct diagnosis is critical. For example, Interferon beta treatment, commonly used for treating MS can worsen the disease outcome of NMOSD patients [48]. 

As the presence of AQP4-IgG is an important biomarker for NMOSD, which can predict disease development [46,47] and allows faster initiation of appropriate treatment, there has been a strong focus on assay development [49]. AQP4-IgG antibodies are detected with highest sensitivity and specificity using a live cell based assay [50,51] in which human embryonic kidney cells (HEK cells) are transfected to express a fluorochrome-tagged M23 AQP4 on their cell surface to closely resemble the native conformation of AQP4 on astrocytes. Serum from patients is then incubated with the cells, and co-localization of human IgG and AQP4 is assessed [29,52]. 

Cell based assays are now considered the gold standard for AQP4-IgG testing [29,30,31,32,53]. AQP4 antibodies target primarily the extracellular part of the M23-AQP4 isoform rather than the extracellular part of full length M1 AQP4 isoform [53,54]. This is associated with the ability of M23 AQP4 to form OAP. Patients’ antibodies bind with high affinity to the OAP, as has been shown for monoclonal AQP4-IgG as well as for AQP4-IgG positive serum samples [29,55]. Fryer and colleagues noted that in flow cytometry assay, nonspecific binding was observed with some samples when cells were transfected with M23-AQP4, but not when transfected with a combination of M1/M23 AQP4 [56]. Nevertheless, cells transfected with M23-AQP4 are considered to detect the presence of AQP4-IgG in serum with high sensitivity and specificity [29,49]. 

It is suggested that the higher binding affinity of AQP4-IgG to OAP is associated with structural changes in the AQP4 epitope upon array assembly [18] or enhanced avidity binding to a multivalent antigen [57].

It is not clear whether the antibodies by themselves are sufficient to cause pathology, or whether ongoing inflammation is required. CNS reactive T cells may be important to breach the BBB and allow antibody entry into the brain [58]. In rodent models of NMOSD, encephalitogenic CNS reactive T cells are used to open the BBB and to allow human AQP4-IgG to penetrate the brain [59,60,61,62]. It has also been suggested that pathogenic T cells may not only be involved in opening the BBB, but contribute to disease pathogenesis since AQP4 reactive T cells that were generated in AQP4 knockout mice result in NMOSD like lesion when transferred to wild type mice [63].

**Figure 1:**
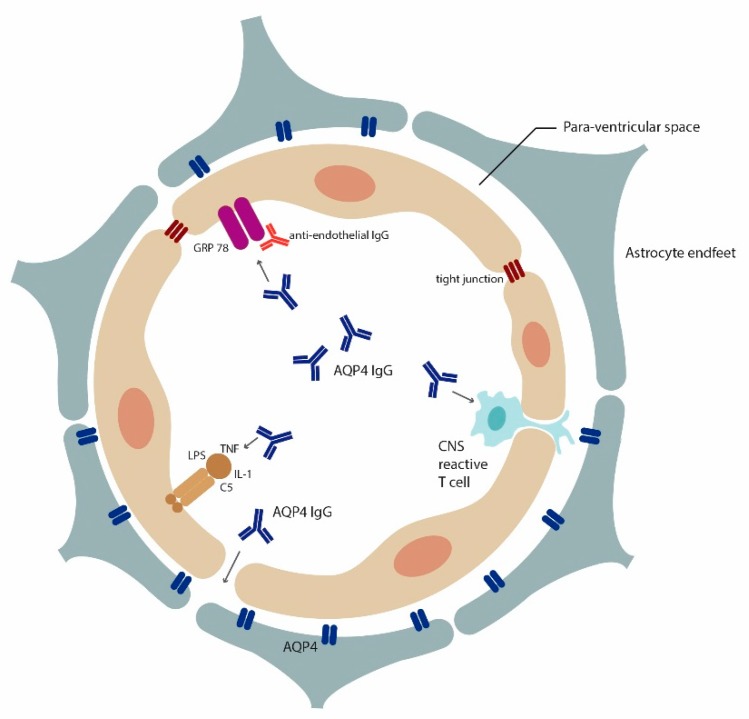
Multiple mechanisms for AQP4-IgG access to the brain: Insights from rodent models. Cross section of a blood vessel demonstrating how AQP4-IgG can enter the brain according to findings from rodent models. BBB breach can occur through encephalitogenic CNS reactive T cells [59,60], inflammatory agents [67] (LPS, TNF alpha, IL-1) and antibodies that alter endothelial cells functional (e.g., anti-GRP78 antibodies [64]). Recently, it was postulated that high affinity AQP4-IgG could enter the brain through circumventricular organs and meningeal or even parenchymal blood vessels without prior BBB insult [65].

It has also been proposed that patients with NMOSD have, in addition to anti-AQP4 antibodies, anti-endothelial antibodies that can breach the BBB [64]. Using a novel proteomic approach investigators showed that brain microvasculature endothelial antibodies target glucose regulated protein 78 (GRP78). Repeated systemic injection of rodents with patient derived monoclonal antibodies to GRP78 breaches the BBB and allows AQP4-IgG to enter the CNS. This would suggest that AQP4-IgG can be pathogenic to the brain, even in absence of pathogenic T cells or BBB insults such as infection. A recent study demonstrated for the first time that a circulating rodent high affinity anti-AQP4 monoclonal antibody that was injected into rats can enter the CNS by itself and cause tissue destruction without addition of pathogenic T cells or active complement [65]. However, this has not been shown for human derived AQP4-IgG so far, and so may be limited to few antibodies or to an unidentified aspect of the experimental protocol. Another study showed that AQP4-IgG binding to astrocytes induces Interleukin-6 (IL6) production, which might decrease BBB function, increase chemokine production and result in increased leukocyte transmigration, and a feed-forward pathologic process (Figure 1) [66].

Several mechanisms can account for the tissue damage following the binding of AQP4-IgG to AQP4 expressed in the brain. The mechanism for which there is most evidence is complement dependent cytotoxicity (CDC) [68,69], in which AQP4-IgG activates complement, leading to irreversible astrocyte loss through the formation of the membrane attack complex (MAC). This mechanism is supported by the fact that most AQP4-IgG are IgG1 [69], which is potent at complement activation. Complement deposition products are indeed present in the brain of NMOSD patients [70], and early clinical trials suggest a benefit of complement inhibitors in NMOSD [71].

Some studies have suggested that rodent complement, particularly mouse, may not be sufficient to activate human antibody mediated CDC [72,73]. Human AQP4-IgG that was directly injected into the brain of rodents required the addition of human complement to induce pathogenicity [73]. However, one has to keep in mind that under quiescent conditions, such as in models in which antibody is injected directly to the brain, there may be less complement in the brain parenchyma than in NMOSD patients [74] with a BBB breach or under inflammatory conditions. 

New evidence suggests that AQP4-IgG can also induce complement independent pathologies [65,67,68,75]. Brain lesions, with or without complement deposition, can be found in NMOSD patients, even within the same patient, suggesting that different mechanisms of AQP4-IgG may act concurrently. Little is known about the mechanism by which AQP4-IgG mediates brain lesions in the absence of complement activation [75]. An in vitro study of cultured human astrocytes provided evidence that both complement dependent and independent astrocytopathy can occur as AQP4-IgG may decrease antigen density in the absence of complement, which could be partially reversed by the removal of IgG from the culture [68]. This may alter astrocyte function. In an in vivo study, IgG from an NMOSD patient was passively transferred to mice with a compromised BBB by prior LPS injection. The mice showed AQP4 astrocyte loss in the spinal cord in the absence of complement activation [67]. There was a massive increase of Iba1 positive microglial cells found in close proximity to the astrocytes [67]. This study proposed a mechanism in which AQP4-IgG acts through antibody dependent cell mediated cytotoxicity (ADCC) by activating microglia expressing FcR including FcγRI, FcγRII, and FcγRIII. Interestingly, anti-AQP4-IgG engineered to have nine fold stronger CDC, but no ADCC, produced much less pathology than wildtype AQP4-IgG in one study [76].

Understanding the mechanisms of tissue destruction caused by AQP4-IgG will pave the way for therapeutic intervention. To date most patients with NMOSD receive immunosuppressive medications. Acute symptoms are commonly treated with Methylprednisolone or plasma exchange [71,77,78]. As preventative therapy, the monoclonal antibody Rituximab, targeting CD20 positive B cells [71], but not antibody secreting plasma cells is commonly used. Patients show improvement with Rituximab therapy [79], despite persistent AQP4-IgG levels [80,81]. Since AQP4-IgG persist following rituximab and patients show beneficial effect, the question remains if AQP4-IgG by itself can cause damage. It is possible that Rituximab may have a different effect, such as depleting B cells that present antigen to pathogenic T cells, or increasing T regulatory cells (Treg) [82,83].

Blocking the binding of pathogenic AQP4-IgG through administration of non-pathogenic AQP4-IgG that lacks CDC and ADCC is emerging as potential therapeutic intervention in NMOSD [84], which would allow avoiding immunosuppressive therapy. If, however, AQP4-IgG acts also through mechanisms other than CDC or ADCC, this type of treatment may not be sufficient to mitigate all pathology. The alternative would be to administer peptide mimeotopes to block pathogenic AQP4-IgG. Such an approach was been suggested for neutralization of brain-reactive antibodies in Neurospsychiatric Systemic Lupus Erythematosus. Blocking the pathogenic epitope may be a challenge as, however there may be multiple epitopes targeted by patients’ antibodies [85].

### 4.2. Role of AQP4 in Controlling Brain-Water Balance

#### AQP4 and Hydrocephalus

Hydrocephalus is a pathologic condition in which increased CSF accumulation in the ventricles often results in increased pressure. Untreated hydrocephalus can lead to brain swelling, brain damage and death. Hydrocephalus is usually caused by impaired clearance of CSF outflow from the brain into the circulatory system. 

Animal models have implicated AQP4 in both hydrocephalus initiation and resolution. The role of AQP4 depends on the disease stage and brain region. In models that use kaolin to induce acute hydrocephalus, there is an increase of AQP4 expression [86,87]. In a rodent model where hydrocephalus is induced by l-alysophosphatidylcholinestearoyl injection, upregulation in AQP4 expression in astrocytes, both on astrocytic endfeet and on the entire membrane of the astrocyte was recorded [88]. AQP4 deficient mice exhibit a sporadic rate of spontaneous hydrocephalus [17] and kaolin induced hydrocephalus in AQP4 deficient mice results in an accelerated progression of hydrocephalus [89]. In patients with congenital hydrocephalus, AQP4, measured by Western blot and ELISA, is elevated in CSF and in the parenchyma [90]. In addition, hydrocephalus has been reported in AQP4-IgG seropositive NMOSD patients [91] as well as vasogenic edema that manifests as posterior reversible encephalopathy syndrome [92]. 

While it is clear that AQP4 has a role in hydrocephalus, it remains unclear whether AQP4 has a protective or deleterious effect. It may contribute to hydrocephalus development at the initial disease stage or be important for edema resolution at a later time point. In addition, AQP4 is considered to have different roles for cytotoxic brain edema, vasogenic brain edema, and hydrocephalus, which may reflect to the different sites where water is accumulated in these conditions. 

This needs to be resolved before addressing a potential treatment approach that would limit water flow into the brain and accelerate outflow into the systemic circulation by modulating AQP4 and might diminish the need for a surgical intervention. 

### 4.3. Role of AQP4 in Glymphatic Clearance, Synaptic Plasticity and Memory

#### AQP4 and Alzheimer Diseases 

**Figure 2:**
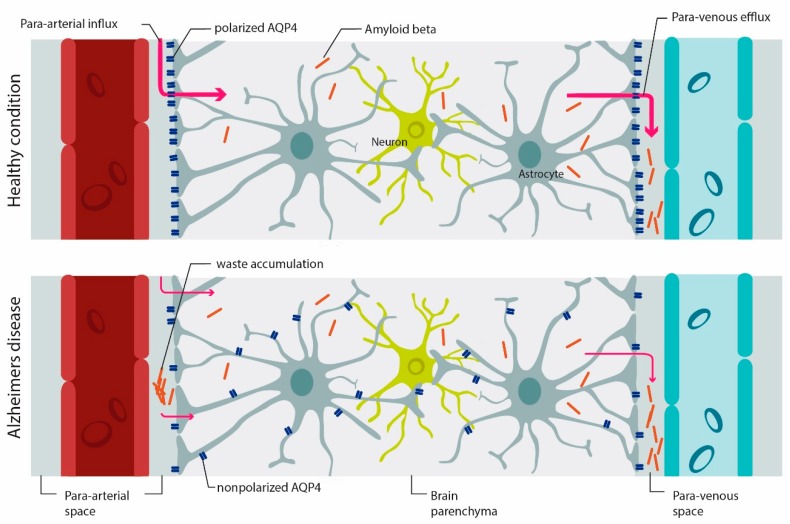
AQP4 mediates-waste clearance through the glymphatic system. In the healthy brain, AQP4 is mainly expressed on astrocyte endfeet (polarized AQP4 expression). CSF circulates through the para-arterial system (para-arterial influx) into the brain parenchyma and then into the veins (para-arterial efflux). With aging, and even more so under pathologic conditions, AQP4 polarization is reduced and there is more expression of AQP4 on parenchymal processes (AQP4 depolarization), which affects the efficiency of the glymphatic system in waste clearance such as beta-amyloid. Accumulation of beta-amyloid is a hallmark of AD [98]. Image modified from [100,101].

With the elucidation of the glymphatic (glial lymphatic) system in 2012 as a system of waste clearance for the brain [93], several studies have associated a breakdown of the glymphatic pathway in different neurodegenerative diseases including AD (Figure 2) [94]. It is believed that the recirculation of CSF flow is driven by active fluid transport through the brain parenchyma from para-arterial to para-venous spaces and that AQP4 supports the clearance of interstitial solutes including beta-amyloid and tau to prevent their aggregation. Accumulation of beta-amyloid is a hallmark of AD and can begin years before disease onset [95]. The glymphatic system is proposed to be most effective during sleep and dysfunctional sleep is further associated with beta-amyloid accumulation [93,96]. It has been shown that glymphatic function is reduced in the mid- to late-stage of AD due to the loss of polarity of AQP4 at the astrocyte endfeet [13]. A recent study suggested that people with certain genetic variations in AQP4 who have poor sleep patterns have more beta-amyloid deposition in the brain due to a dysfunctional glymphatic system [97]. The role of AQP4 in the glymphatic system should be further investigated [98]. 

AQP4 is also implicated in regulating glutamate transporter-1 (GLT-1) function. Astrocytes in AQP4 deficient mice have reduced GLT-1 levels as well as reduced glutamate clearance, which in turns affects synaptic plasticity and memory since function can be rescued by a potent GLT-1 stimulator [99]. This further emphasizes that AQP4 may be an interesting and novel target in many diseases.

## 5. Conclusions

Alterations in AQP4 expression or loss of polarization, or both, is now reported in many diseases (Table 1). In some conditions, such as hydrocephalus and stroke, the role of AQP4 has been studied for some time, while, in other diseases such as AD the role for AQP4 emerged only recently.

AQP4 function, particularly regarding its role in brain water balance, may differ in different brain areas. Moreover the consequence of AQP4 dysfunction may depend on the stage of the disease that is being studied. Rodent models trying to elucidate the role of AQP4 in different disease models can therefore be conflicting. Moreover, it is emerging that AQP4 helps regulate glutamate levels and GLT-1 expression [102]. More will be learned about changes in brain homeostasis following dysfunction of AQP4 in the coming years. 

AQP4 inhibitors or down regulators may provide future potential treatment of some conditions such as for cytotoxic brain edema [103]. 2-(nicotinamide)-1,3,4-thiadiazole (TGN-020) was shown to serve as a potent AQP4 inhibitor in an ischemic rodent stroke model [104]. In contrast, enhancers of AQP4 expression may be beneficial in reducing vasogenic brain swelling.

Therapeutic strategies need to address access of AQP4 modulators across a functionally intact BBB, since not all pathological conditions are associated with impaired BBB. Aquaporumab is an example of an AQP4-targeted antibody therapeutics [84]. This is a non-pathogenic- AQP4-IgG lacking CDC and ADCC, so it can bind AQP4 without causing NMO lesions, blocking the ability of pathogenic AQP4-IgG to bind. AQP4 blocking antibodies as well as AQP4-IgG enzymatic inactivation are in preclinical development, and it will be important to determine when and where it traverses the BBB.

It is abundantly clear that the functions of AQP4 in the CNS are more diverse and more complex than previously appreciated. Targeted therapeutics with maximum benefit and limited toxicity will depend on a thorough understanding of the biology or AQP4 in health and in the myriad pathologic conditions that we know include aberrant AQP4 expression.

## Figures and Tables

**Table 1:** AQP4 in different pathological conditions.

Disease	Mechanism	AQP4 Related Pathology	Rodent Models	(Potential) Therapeutic
**NMOSD**	-AQP4-IgG dependent tissue astrocytophathy: -CDC -ADCC -other mechanisms	-Presence of AQP4-IgG in serum [44] -Loss of AQP4 in NMOSD lesions [105] -Massive demyelination (brain, optic nerve, spinal cord) [70,75]	1. Injection of anti-AQP4-IgG into the brain [73] 2. Intravenous injection of AQP4-IgG following BBB disruption through T cell- mediated EAE [59,60], bacterial proteins [67] or anti-endothelial antibody [64]. 3. High affinity circulating rodent AQP4-IgG enter the CNS without BBB impairment [65]	-Decoy antibody lacking FcR or complement binding [84] -Recombinant IgG1 Fc hexamers that block cytotoxicity and pathological changes [106]
**Alzheimer’s Disease**	Loss of AQP4 polarization with impaired clearance of interstitial solutes and increased aggregation (beta- amyloid)	Mislocalization of AQP4 [13]	AQP4 gene knockout of beta-amyloid precursor protein /presenilin 1 (APP/PS1) transgenic mice [107]	AQP4 receptor agonists [107]
**ALS**	Loss of AQP4 polarization and altered AQP4 expression is contributing to motor neuron degeneration [107] and BBB impairment [108]	AQP4 overexpression in astrocytes [109]	Superoxide dismutase 1 (SOD1) G93A transgenic mice (mouse model of ALS) [109]	Targeting AQP4 as potential treatment to restore BBB in ALS [108]
**Parkinson’s Disease (PD)**	AQP4 dysfunction contributing to synuclein deposition and water accumulation in the substantia nigra [110]	Enriched AQP4-positive astrocytes in the neocortex [111]	AQP4 deficient mice treated with MPTP [112]	N/A
**Ischemic Stroke**	AQP4 enhances edema formation or diminishes resolution	Enhanced expression of AQP4 at site of infarction [10].	Brain edema caused by acute water intoxication using AQP4 knock out mice [41]	AQP4 inhibitors during edema formation
**Traumatic brain injury (TBI)**	AQP4 is altering water homeostasis and AQP4 may be associated with neuroinflammation (through astrocyte and microglia activation)	Increased expression of AQP4 and loss of AQP4 polarity [9]	TBI mouse model [9]	AQP4 inhibitors may be beneficial [103]
**Hydrocephalus**	Control of water homeostasis	-Increased AQP4 in CSF of congenital communicating hydrocephalus [90], -Hydrocephalus has been reported in AQP4-IgG positive NMOSD [91]	Rat kaolin model [86]	Increasing AQP4 to support CSF clearance at a later disease stage or decreasing AQP4 in areas of CSF production particularly at disease onset [113].
**Glioma**	-AQP4 is contributing to increased tumor cell migration possibly through increasing water permeability [114]. -Involvement of AQP4 in tumor edema	Expression of AQP4 in human glioblastoma [115]	N/A	Use of AQP inhibitors to reduce tumor growth [18]
**Schizophrenia**	Reduced AQP4 is contributing to neurovascular dysfunction and BBB hyperpermeability	Astroglial loss and reduced AQP4 expression in the deep layers of the anterior cingulated gyrus [116]	N/A	N/A
**Major depressive disorder (MDD)**	AQP4 is contributing to poor water balance	Reduced coverage of blood vessels by AQP4 positive astrocytic endfeet in the orbitofrontal cortex [117]	N/A	N/A
**Epilepsy**	Impairment of K+ homeostasis	AQP4 expression is increased in samples from atrophic hippocampus from epileptic patients [118]	AQP4 deficient mice [119]	AQP4 modulators to increase seizure thresholds [103]
**Autism**	Abnormal glial-neuronal communication in brains of subjects with autism	Decreased AQP4 expression in cerebellum of post mortem tissue [120]	N/A	N/A

## References

[1] Badaut J., Brunet J.F., Regli L. (2007). Aquaporins in the brain: From aqueduct to “multi-duct”. Metab. Brain Dis..

[2] Rash J.E., Yasumura T., Hudson C.S., Agre P., Nielsen S. (1998). Direct immunogold labeling of aquaporin-4 in square arrays of astrocyte and ependymocyte plasma membranes in rat brain and spinal cord. Proc. Natl. Acad. Sci. USA.

[3] Nielsen S., Nagelhus E.A., Amiry-Moghaddam M., Bourque C., Agre P., Ottersen O.P. (1997). Specialized membrane domains for water transport in glial cells: High-resolution immunogold cytochemistry of aquaporin-4 in rat brain. J. Neurosci..

[4] Hasegawa H., Ma T., Skach W., Matthay M.A., Verkman A.S. (1994). Molecular cloning of a mercurial-insensitive water channel expressed in selected water-transporting tissues. J. Biol. Chem..

[5] Jung J.S., Bhat R.V., Preston G.M., Guggino W.B., Baraban J.M., Agre P. (1994). Molecular characterization of an aquaporin cDNA from brain: Candidate osmoreceptor and regulator of water balance. Proc. Natl. Acad. Sci. USA.

[6] Murlidharan G., Crowther A., Reardon R.A., Song J., Asokan A. (2016). Glymphatic fluid transport controls paravascular clearance of AAV vectors from the brain. JCI Insight.

[7] Verkman A.S., Binder D.K., Bloch O., Auguste K., Papadopoulos M.C. (2006). Three distinct roles of aquaporin-4 in brain function revealed by knockout mice. Biochim. Biophys. Acta.

[8] Nagelhus E.A., Ottersen O.P. (2013). Physiological roles of aquaporin-4 in brain. Physiol. Rev..

[9] Zhao Z.A., Li P., Ye S.Y., Ning Y.L., Wang H., Peng Y., Yang N., Zhao Y., Zhang Z.H., Chen J.F. (2017). Perivascular AQP4 dysregulation in the hippocampal CA1 area after traumatic brain injury is alleviated by adenosine A2A receptor inactivation. Sci. Rep..

[10] Aoki K., Uchihara T., Tsuchiya K., Nakamura A., Ikeda K., Wakayama Y. (2003). Enhanced expression of aquaporin 4 in human brain with infarction. Acta Neuropathol..

[11] Hubbard J.A., Szu J.I., Binder D.K. (2018). The role of aquaporin-4 in synaptic plasticity, memory and disease. Brain Res. Bull..

[12] Papadopoulos M.C., Verkman A.S. (2012). Aquaporin 4 and neuromyelitis optica. Lancet Neurol..

[13] Zeppenfeld D.M., Simon M., Haswell J.D., D’Abreo D., Murchison C., Quinn J.F., Grafe M.R., Woltjer R.L., Kaye J., Iliff J.J. (2017). Association of Perivascular Localization of Aquaporin-4 With Cognition and Alzheimer Disease in Aging Brains. JAMA Neurol..

[14] Reemst K., Noctor S.C., Lucassen P.J., Hol E.M. (2016). The Indispensable Roles of Microglia and Astrocytes during Brain Development. Front. Hum. Neurosci..

[15] Aprea J., Calegari F. (2015). Long non-coding RNAs in corticogenesis: Deciphering the non-coding code of the brain. EMBO J..

[16] Fallier-Becker P., Vollmer J.P., Bauer H.C., Noell S., Wolburg H., Mack A.F. (2014). Onset of aquaporin-4 expression in the developing mouse brain. Int. J. Dev. Neurosci..

[17] Feng X., Papadopoulos M.C., Liu J., Li L., Zhang D., Zhang H., Verkman A.S., Ma T. (2009). Sporadic obstructive hydrocephalus in Aqp4 null mice. J. Neurosci. Res..

[18] Papadopoulos M.C., Verkman A.S. (2013). Aquaporin water channels in the nervous system. Nat. Rev. Neurosci..

[19] Walz T., Fujiyoshi Y., Engel A. (2009). The AQP structure and functional implications. Handb. Exp. Pharmacol..

[20] Ho J.D., Yeh R., Sandstrom A., Chorny I., Harries W.E., Robbins R.A., Miercke L.J., Stroud R.M. (2009). Crystal structure of human aquaporin 4 at 1.8 A and its mechanism of conductance. Proc. Natl. Acad. Sci. USA.

[21] Lu M., Lee M.D., Smith B.L., Jung J.S., Agre P., Verdijk M.A., Merkx G., Rijss J.P., Deen P.M. (1996). The human AQP4 gene: Definition of the locus encoding two water channel polypeptides in brain. Proc. Natl. Acad. Sci. USA.

[22] Rossi A., Moritz T.J., Ratelade J., Verkman A.S. (2012). Super-resolution imaging of aquaporin-4 orthogonal arrays of particles in cell membranes. J. Cell Sci..

[23] Furman C.S., Gorelick-Feldman D.A., Davidson K.G., Yasumura T., Neely J.D., Agre P., Rash J.E. (2003). Aquaporin-4 square array assembly: Opposing actions of M1 and M23 isoforms. Proc. Natl. Acad. Sci. USA.

[24] Crane J.M., Bennett J.L., Verkman A.S. (2009). Live cell analysis of aquaporin-4 m1/m23 interactions and regulated orthogonal array assembly in glial cells. J. Biol. Chem..

[25] Yang B., van Hoek A.N., Verkman A.S. (1997). Very high single channel water permeability of aquaporin-4 in baculovirus-infected insect cells and liposomes reconstituted with purified aquaporin-4. Biochemistry.

[26] Hiroaki Y., Tani K., Kamegawa A., Gyobu N., Nishikawa K., Suzuki H., Walz T., Sasaki S., Mitsuoka K., Kimura K. (2006). Implications of the aquaporin-4 structure on array formation and cell adhesion. J. Mol. Biol..

[27] Silberstein C., Bouley R., Huang Y., Fang P., Pastor-Soler N., Brown D., Van Hoek A.N. (2004). Membrane organization and function of M1 and M23 isoforms of aquaporin-4 in epithelial cells. Am. J. Physiol. Renal Physiol..

[28] Verkman A.S., Ratelade J., Rossi A., Zhang H., Tradtrantip L. (2011). Aquaporin-4: Orthogonal array assembly, CNS functions, and role in neuromyelitis optica. Acta Pharmacol. Sin..

[29] Mader S., Lutterotti A., Di Pauli F., Kuenz B., Schanda K., Aboul-Enein F., Khalil M., Storch M.K., Jarius S., Kristoferitsch W. (2010). Patterns of antibody binding to aquaporin-4 isoforms in neuromyelitis optica. PLoS ONE.

[30] Nicchia G.P., Mastrototaro M., Rossi A., Pisani F., Tortorella C., Ruggieri M., Lia A., Trojano M., Frigeri A., Svelto M. (2009). Aquaporin-4 orthogonal arrays of particles are the target for neuromyelitis optica autoantibodies. Glia.

[31] Crane J.M., Lam C., Rossi A., Gupta T., Bennett J.L., Verkman A.S. (2011). Binding affinity and specificity of neuromyelitis optica autoantibodies to aquaporin-4 M1/M23 isoforms and orthogonal arrays. J. Biol. Chem..

[32] Miyazaki K., Abe Y., Iwanari H., Suzuki Y., Kikuchi T., Ito T., Kato J., Kusano-Arai O., Takahashi T., Nishiyama S. (2013). Establishment of monoclonal antibodies against the extracellular domain that block binding of NMO-IgG to AQP4. J. Neuroimmunol..

[33] Neely J.D., Amiry-Moghaddam M., Ottersen O.P., Froehner S.C., Agre P., Adams M.E. (2001). Syntrophin-dependent expression and localization of Aquaporin-4 water channel protein. Proc. Natl. Acad. Sci. USA.

[34] Saadoun S., Papadopoulos M.C., Davies D.C., Krishna S., Bell B.A. (2002). Aquaporin-4 expression is increased in oedematous human brain tumours. J. Neurol. Neurosurg. Psychiatry.

[35] Solenov E., Watanabe H., Manley G.T., Verkman A.S. (2004). Sevenfold-reduced osmotic water permeability in primary astrocyte cultures from AQP-4-deficient mice, measured by a fluorescence quenching method. Am. J. Physiol. Cell Physiol..

[36] Haj-Yasein N.N., Vindedal G.F., Eilert-Olsen M., Gundersen G.A., Skare O., Laake P., Klungland A., Thoren A.E., Burkhardt J.M., Ottersen O.P. (2011). Glial-conditional deletion of aquaporin-4 (Aqp4) reduces blood-brain water uptake and confers barrier function on perivascular astrocyte endfeet. Proc. Natl. Acad. Sci. USA.

[37] Skucas V.A., Mathews I.B., Yang J., Cheng Q., Treister A., Duffy A.M., Verkman A.S., Hempstead B.L., Wood M.A., Binder D.K. (2011). Impairment of select forms of spatial memory and neurotrophin-dependent synaptic plasticity by deletion of glial aquaporin-4. J. Neurosci..

[38] Zhang J., Li Y., Chen Z.G., Dang H., Ding J.H., Fan Y., Hu G. (2013). Glia protein aquaporin-4 regulates aversive motivation of spatial memory in Morris water maze. CNS Neurosci. Ther..

[39] Zhou J., Kong H., Hua X., Xiao M., Ding J., Hu G. (2008). Altered blood-brain barrier integrity in adult aquaporin-4 knockout mice. Neuroreport.

[40] Saadoun S., Tait M.J., Reza A., Davies D.C., Bell B.A., Verkman A.S., Papadopoulos M.C. (2009). AQP4 gene deletion in mice does not alter blood-brain barrier integrity or brain morphology. Neuroscience.

[41] Manley G.T., Fujimura M., Ma T., Noshita N., Filiz F., Bollen A.W., Chan P., Verkman A.S. (2000). Aquaporin-4 deletion in mice reduces brain edema after acute water intoxication and ischemic stroke. Nat. Med..

[42] Papadopoulos M.C., Manley G.T., Krishna S., Verkman A.S. (2004). Aquaporin-4 facilitates reabsorption of excess fluid in vasogenic brain edema. FASEB J..

[43] Lennon V.A., Wingerchuk D.M., Kryzer T.J., Pittock S.J., Lucchinetti C.F., Fujihara K., Nakashima I., Weinshenker B.G. (2004). A serum autoantibody marker of neuromyelitis optica: Distinction from multiple sclerosis. Lancet.

[44] Lennon V.A., Kryzer T.J., Pittock S.J., Verkman A.S., Hinson S.R. (2005). IgG marker of optic-spinal multiple sclerosis binds to the aquaporin-4 water channel. J. Exp. Med..

[45] Jarius S., Probst C., Borowski K., Franciotta D., Wildemann B., Stoecker W., Wandinger K.P. (2010). Standardized method for the detection of antibodies to aquaporin-4 based on a highly sensitive immunofluorescence assay employing recombinant target antigen. J. Neurol. Sci..

[46] Weinshenker B.G., Wingerchuk D.M., Vukusic S., Linbo L., Pittock S.J., Lucchinetti C.F., Lennon V.A. (2006). Neuromyelitis optica IgG predicts relapse after longitudinally extensive transverse myelitis. Ann. Neurol..

[47] Wingerchuk D.M., Banwell B., Bennett J.L., Cabre P., Carroll W., Chitnis T., de Seze J., Fujihara K., Greenberg B., Jacob A. (2015). International consensus diagnostic criteria for neuromyelitis optica spectrum disorders. Neurology.

[48] Palace J., Leite M.I., Nairne A., Vincent A. (2010). Interferon Beta treatment in neuromyelitis optica: Increase in relapses and aquaporin 4 antibody titers. Arch Neurol..

[49] Waters P., Reindl M., Saiz A., Schanda K., Tuller F., Kral V., Nytrova P., Sobek O., Nielsen H.H., Barington T. (2016). Multicentre comparison of a diagnostic assay: Aquaporin-4 antibodies in neuromyelitis optica. J. Neurol. Neurosurg. Psychiatry.

[50] Waters P.J., McKeon A., Leite M.I., Rajasekharan S., Lennon V.A., Villalobos A., Palace J., Mandrekar J.N., Vincent A., Bar-Or A. (2012). Serologic diagnosis of NMO: A multicenter comparison of aquaporin-4-IgG assays. Neurology.

[51] Takahashi T., Fujihara K., Nakashima I., Misu T., Miyazawa I., Nakamura M., Watanabe S., Shiga Y., Kanaoka C., Fujimori J. (2007). Anti-aquaporin-4 antibody is involved in the pathogenesis of NMO: A study on antibody titre. Brain.

[52] Mader S., Gredler V., Schanda K., Rostasy K., Dujmovic I., Pfaller K., Lutterotti A., Jarius S., Di Pauli F., Kuenz B. (2011). Complement activating antibodies to myelin oligodendrocyte glycoprotein in neuromyelitis optica and related disorders. J. Neuroinflamm..

[53] Kitley J., Woodhall M., Leite M.I., Palace J., Vincent A., Waters P. (2015). Aquaporin-4 antibody isoform binding specificities do not explain clinical variations in NMO. Neurol. Neuroimmunol. Neuroinflamm..

[54] Di Pauli F., Mader S., Rostasy K., Schanda K., Bajer-Kornek B., Ehling R., Deisenhammer F., Reindl M., Berger T. (2011). Temporal dynamics of anti-MOG antibodies in CNS demyelinating diseases. Clin. Immunol..

[55] Verkman A.S., Phuan P.W., Asavapanumas N., Tradtrantip L. (2013). Biology of AQP4 and anti-AQP4 antibody: Therapeutic implications for NMO. Brain Pathol..

[56] Fryer J.P., Lennon V.A., Pittock S.J., Jenkins S.M., Fallier-Becker P., Clardy S.L., Horta E., Jedynak E.A., Lucchinetti C.F., Shuster E.A. (2014). AQP4 autoantibody assay performance in clinical laboratory service. Neurol. Neuroimmunol. Neuroinflamm..

[57] Huang P., Takai Y., Kusano-Arai O., Ramadhanti J., Iwanari H., Miyauchi T., Sakihama T., Han J.Y., Aoki M., Hamakubo T. (2016). The binding property of a monoclonal antibody against the extracellular domains of aquaporin-4 directs aquaporin-4 toward endocytosis. Biochem. Biophys. Rep..

[58] Bradl M., Lassmann H. (2010). Oligodendrocytes: Biology and pathology. Acta Neuropathol..

[59] Bradl M., Misu T., Takahashi T., Watanabe M., Mader S., Reindl M., Adzemovic M., Bauer J., Berger T., Fujihara K. (2009). Neuromyelitis optica: Pathogenicity of patient immunoglobulin in vivo. Ann. Neurol..

[60] Bennett J.L., Lam C., Kalluri S.R., Saikali P., Bautista K., Dupree C., Glogowska M., Case D., Antel J.P., Owens G.P. (2009). Intrathecal pathogenic anti-aquaporin-4 antibodies in early neuromyelitis optica. Ann. Neurol..

[61] Pohl M., Kawakami N., Kitic M., Bauer J., Martins R., Fischer M.T., Machado-Santos J., Mader S., Ellwart J.W., Misu T. (2013). T cell-activation in neuromyelitis optica lesions plays a role in their formation. Acta Neuropathol. Commun..

[62] Pohl M., Fischer M.T., Mader S., Schanda K., Kitic M., Sharma R., Wimmer I., Misu T., Fujihara K., Reindl M. (2011). Pathogenic T cell responses against aquaporin 4. Acta Neuropathol..

[63] Jones M.V., Huang H., Calabresi P.A., Levy M. (2015). Pathogenic aquaporin-4 reactive T cells are sufficient to induce mouse model of neuromyelitis optica. Acta Neuropathol. Commun..

[64] Shimizu F., Schaller K.L., Owens G.P., Cotleur A.C., Kellner D., Takeshita Y., Obermeier B., Kryzer T.J., Sano Y., Kanda T. (2017). Glucose-regulated protein 78 autoantibody associates with blood-brain barrier disruption in neuromyelitis optica. Sci. Transl. Med..

[65] Hillebrand S., Schanda K., Nigritinou M., Tsymala I., Bohm D., Peschl P., Takai Y., Fujihara K., Nakashima I., Misu T. (2018). Circulating AQP4-specific auto-antibodies alone can induce neuromyelitis optica spectrum disorder in the rat. Acta Neuropathol..

[66] Takeshita Y., Obermeier B., Cotleur A.C., Spampinato S.F., Shimizu F., Yamamoto E., Sano Y., Kryzer T.J., Lennon V.A., Kanda T. (2017). Effects of neuromyelitis optica-IgG at the blood-brain barrier in vitro. Neurol. Neuroimmunol. Neuroinflamm..

[67] Yick L.W., Ma O.K., Ng R.C., Kwan J.S., Chan K.H. (2018). Aquaporin-4 Autoantibodies from Neuromyelitis Optica Spectrum Disorder Patients Induce Complement-Independent Immunopathologies in Mice. Front. Immunol..

[68] Nishiyama S., Misu T., Nuriya M., Takano R., Takahashi T., Nakashima I., Yasui M., Itoyama Y., Aoki M., Fujihara K. (2016). Complement-dependent and -independent aquaporin 4-antibody-mediated cytotoxicity in human astrocytes: Pathogenetic implications in neuromyelitis optica. Biochem. Biophys. Rep..

[69] Hinson S.R., Pittock S.J., Lucchinetti C.F., Roemer S.F., Fryer J.P., Kryzer T.J., Lennon V.A. (2007). Pathogenic potential of IgG binding to water channel extracellular domain in neuromyelitis optica. Neurology.

[70] Lucchinetti C.F., Mandler R.N., McGavern D., Bruck W., Gleich G., Ransohoff R.M., Trebst C., Weinshenker B., Wingerchuk D., Parisi J.E. (2002). A role for humoral mechanisms in the pathogenesis of Devic’s neuromyelitis optica. Brain.

[71] Papadopoulos M.C., Bennett J.L., Verkman A.S. (2014). Treatment of neuromyelitis optica: State-of-the-art and emerging therapies. Nat. Rev. Neurol..

[72] Bergman I., Basse P.H., Barmada M.A., Griffin J.A., Cheung N.K. (2000). Comparison of in vitro antibody-targeted cytotoxicity using mouse, rat and human effectors. Cancer Immunol. Immunother..

[73] Saadoun S., Waters P., Bell B.A., Vincent A., Verkman A.S., Papadopoulos M.C. (2010). Intra-cerebral injection of neuromyelitis optica immunoglobulin G and human complement produces neuromyelitis optica lesions in mice. Brain.

[74] Nytrova P., Potlukova E., Kemlink D., Woodhall M., Horakova D., Waters P., Havrdova E., Zivorova D., Vincent A., Trendelenburg M. (2014). Complement activation in patients with neuromyelitis optica. J. Neuroimmunol..

[75] Misu T., Hoftberger R., Fujihara K., Wimmer I., Takai Y., Nishiyama S., Nakashima I., Konno H., Bradl M., Garzuly F. (2013). Presence of six different lesion types suggests diverse mechanisms of tissue injury in neuromyelitis optica. Acta Neuropathol..

[76] Ratelade J., Asavapanumas N., Ritchie A.M., Wemlinger S., Bennett J.L., Verkman A.S. (2013). Involvement of antibody-dependent cell-mediated cytotoxicity in inflammatory demyelination in a mouse model of neuromyelitis optica. Acta Neuropathol..

[77] Trebst C., Jarius S., Berthele A., Paul F., Schippling S., Wildemann B., Borisow N., Kleiter I., Aktas O., Kumpfel T. (2014). Update on the diagnosis and treatment of neuromyelitis optica: Recommendations of the Neuromyelitis Optica Study Group (NEMOS). J. Neurol..

[78] Kleiter I., Gahlen A., Borisow N., Fischer K., Wernecke K.D., Wegner B., Hellwig K., Pache F., Ruprecht K., Havla J. (2016). Neuromyelitis optica: Evaluation of 871 attacks and 1,153 treatment courses. Ann. Neurol..

[79] Cree B.A., Lamb S., Morgan K., Chen A., Waubant E., Genain C. (2005). An open label study of the effects of rituximab in neuromyelitis optica. Neurology.

[80] Gredler V., Mader S., Schanda K., Hegen H., Di Pauli F., Kuenz B., Deisenhammer F., Berger T., Reindl M., Lutterotti A. (2013). Clinical and immunological follow-up of B-cell depleting therapy in CNS demyelinating diseases. J. Neurol. Sci..

[81] Mealy M.A., Kim S.H., Schmidt F., Lopez R., Jimenez Arango J.A., Paul F., Wingerchuk D.M., Greenberg B.M., Kim H.J., Levy M. (2018). Aquaporin-4 serostatus does not predict response to immunotherapy in neuromyelitis optica spectrum disorders. Mult. Scler..

[82] Sfikakis P.P., Souliotis V.L., Fragiadaki K.G., Moutsopoulos H.M., Boletis J.N., Theofilopoulos A.N. (2007). Increased expression of the FoxP3 functional marker of regulatory T cells following B cell depletion with rituximab in patients with lupus nephritis. Clin. Immunol..

[83] Reis E.A., Athanazio D.A., Lima I., Oliveira e Silva N., Andrade J.C., Jesus R.N., Barbosa L.M., Reis M.G., Santiago M.B. (2009). NK and NKT cell dynamics after rituximab therapy for systemic lupus erythematosus and rheumatoid arthritis. Rheumatol. Int..

[84] Tradtrantip L., Zhang H., Saadoun S., Phuan P.W., Lam C., Papadopoulos M.C., Bennett J.L., Verkman A.S. (2012). Anti-aquaporin-4 monoclonal antibody blocker therapy for neuromyelitis optica. Ann. Neurol..

[85] Soltys J.N., Meyer S.A., Schumann H., Gibson E.A., Restrepo D., Bennett J.L. (2017). Determining the Spatial Relationship of Membrane-Bound Aquaporin-4 Autoantibodies by STED Nanoscopy. Biophys. J..

[86] Mao X., Enno T.L., Del Bigio M.R. (2006). Aquaporin 4 changes in rat brain with severe hydrocephalus. Eur. J. Neurosci..

[87] Skjolding A.D., Rowland I.J., Sogaard L.V., Praetorius J., Penkowa M., Juhler M. (2010). Hydrocephalus induces dynamic spatiotemporal regulation of aquaporin-4 expression in the rat brain. Cereb. Fluid Res..

[88] Tourdias T., Dragonu I., Fushimi Y., Deloire M.S., Boiziau C., Brochet B., Moonen C., Petry K.G., Dousset V. (2009). Aquaporin 4 correlates with apparent diffusion coefficient and hydrocephalus severity in the rat brain: A combined MRI-histological study. Neuroimage.

[89] Bloch O., Auguste K.I., Manley G.T., Verkman A.S. (2006). Accelerated progression of kaolin-induced hydrocephalus in aquaporin-4-deficient mice. J. Cereb. Blood Flow Metab..

[90] Castaneyra-Ruiz L., Gonzalez-Marrero I., Gonzalez-Toledo J.M., Castaneyra-Ruiz A., de Paz-Carmona H., Castaneyra-Perdomo A., Carmona-Calero E.M. (2013). Aquaporin-4 expression in the cerebrospinal fluid in congenital human hydrocephalus. Fluids Barriers CNS.

[91] Clardy S.L., Lucchinetti C.F., Krecke K.N., Lennon V.A., O’Toole O., Weinshenker B.G., Boyd C.D., Krieger S., McGraw C., Guo Y. (2014). Hydrocephalus in neuromyelitis optica. Neurology.

[92] Magana S.M., Matiello M., Pittock S.J., McKeon A., Lennon V.A., Rabinstein A.A., Shuster E., Kantarci O.H., Lucchinetti C.F., Weinshenker B.G. (2009). Posterior reversible encephalopathy syndrome in neuromyelitis optica spectrum disorders. Neurology.

[93] Iliff J.J., Wang M., Liao Y., Plogg B.A., Peng W., Gundersen G.A., Benveniste H., Vates G.E., Deane R., Goldman S.A. (2012). A paravascular pathway facilitates CSF flow through the brain parenchyma and the clearance of interstitial solutes, including amyloid beta. Sci. Transl. Med..

[94] Rasmussen M.K., Mestre H., Nedergaard M. (2018). The glymphatic pathway in neurological disorders. Lancet Neurol..

[95] Karran E., Mercken M., De Strooper B. (2011). The amyloid cascade hypothesis for Alzheimer’s disease: An appraisal for the development of therapeutics. Nat. Rev. Drug Discov..

[96] Shokri-Kojori E., Wang G.J., Wiers C.E., Demiral S.B., Guo M., Kim S.W., Lindgren E., Ramirez V., Zehra A., Freeman C. (2018). beta-Amyloid accumulation in the human brain after one night of sleep deprivation. Proc. Natl. Acad. Sci. USA.

[97] Rainey-Smith S.R., Mazzucchelli G.N., Villemagne V.L., Brown B.M., Porter T., Weinborn M., Bucks R.S., Milicic L., Sohrabi H.R., Taddei K. (2018). Genetic variation in Aquaporin-4 moderates the relationship between sleep and brain Abeta-amyloid burden. Transl. Psychiatry.

[98] Smith A.J., Verkman A.S. (2018). The “glymphatic” mechanism for solute clearance in Alzheimer’s disease: Game changer or unproven speculation?. FASEB J..

[99] Yang J., Li M.X., Luo Y., Chen T., Liu J., Fang P., Jiang B., Hu Z.L., Jin Y., Chen J.G. (2013). Chronic ceftriaxone treatment rescues hippocampal memory deficit in AQP4 knockout mice via activation of GLT-1. Neuropharmacology.

[100] Jessen N.A., Munk A.S., Lundgaard I., Nedergaard M. (2015). The Glymphatic System: A Beginner’s Guide. Neurochem. Res..

[101] Louveau A., Plog B.A., Antila S., Alitalo K., Nedergaard M., Kipnis J. (2017). Understanding the functions and relationships of the glymphatic system and meningeal lymphatics. J. Clin. Investig..

[102] Zeng X.N., Sun X.L., Gao L., Fan Y., Ding J.H., Hu G. (2007). Aquaporin-4 deficiency down-regulates glutamate uptake and GLT-1 expression in astrocytes. Mol. Cell. Neurosci..

[103] Papadopoulos M.C., Verkman A.S. (2008). Potential utility of aquaporin modulators for therapy of brain disorders. Prog. Brain Res..

[104] Pirici I., Balsanu T.A., Bogdan C., Margaritescu C., Divan T., Vitalie V., Mogoanta L., Pirici D., Carare R.O., Muresanu D.F. (2017). Inhibition of Aquaporin-4 Improves the Outcome of Ischaemic Stroke and Modulates Brain Paravascular Drainage Pathways. Int. J. Mol. Sci..

[105] Misu T., Fujihara K., Kakita A., Konno H., Nakamura M., Watanabe S., Takahashi T., Nakashima I., Takahashi H., Itoyama Y. (2007). Loss of aquaporin 4 in lesions of neuromyelitis optica: Distinction from multiple sclerosis. Brain.

[106] Tradtrantip L., Felix C.M., Spirig R., Morelli A.B., Verkman A.S. (2018). Recombinant IgG1 Fc hexamers block cytotoxicity and pathological changes in experimental in vitro and rat models of neuromyelitis optica. Neuropharmacology.

[107] Xu Z., Xiao N., Chen Y., Huang H., Marshall C., Gao J., Cai Z., Wu T., Hu G., Xiao M. (2015). Deletion of aquaporin-4 in APP/PS1 mice exacerbates brain Abeta accumulation and memory deficits. Mol. Neurodegener..

[108] Foglio E., Rodella L.F. (2010). Aquaporins and neurodegenerative diseases. Curr. Neuropharmacol..

[109] Watanabe-Matsumoto S., Moriwaki Y., Okuda T., Ohara S., Yamanaka K., Abe Y., Yasui M., Misawa H. (2018). Dissociation of blood-brain barrier disruption and disease manifestation in an aquaporin-4-deficient mouse model of amyotrophic lateral sclerosis. Neurosci. Res..

[110] Prydz A., Stahl K., Puchades M., Davarpaneh N., Nadeem M., Ottersen O.P., Gundersen V., Amiry-Moghaddam M. (2017). Subcellular expression of aquaporin-4 in substantia nigra of normal and MPTP-treated mice. Neuroscience.

[111] Hoshi A., Tsunoda A., Tada M., Nishizawa M., Ugawa Y., Kakita A. (2017). Expression of Aquaporin 1 and Aquaporin 4 in the Temporal Neocortex of Patients with Parkinson’s Disease. Brain Pathol..

[112] Fan Y., Kong H., Shi X., Sun X., Ding J., Wu J., Hu G. (2008). Hypersensitivity of aquaporin 4-deficient mice to 1-methyl-4-phenyl-1,2,3,6-tetrahydropyrindine and astrocytic modulation. Neurobiol. Aging.

[113] Desai B., Hsu Y., Schneller B., Hobbs J.G., Mehta A.I., Linninger A. (2016). Hydrocephalus: The role of cerebral aquaporin-4 channels and computational modeling considerations of cerebrospinal fluid. Neurosurg. Focus.

[114] Tome-Garcia J., Erfani P., Nudelman G., Tsankov A.M., Katsyv I., Tejero R., Bin Z., Walsh M., Friedel R.H., Zaslavsky E. (2018). Analysis of chromatin accessibility uncovers TEAD1 as a regulator of migration in human glioblastoma. Nat. Commun..

[115] Maugeri R., Schiera G., Di Liegro C.M., Fricano A., Iacopino D.G., Di Liegro I. (2016). Aquaporins and Brain Tumors. Int. J. Mol. Sci..

[116] Katsel P., Byne W., Roussos P., Tan W., Siever L., Haroutunian V. (2011). Astrocyte and glutamate markers in the superficial, deep, and white matter layers of the anterior cingulate gyrus in schizophrenia. Neuropsychopharmacology.

[117] Rajkowska G., Hughes J., Stockmeier C.A., Javier Miguel-Hidalgo J., Maciag D. (2013). Coverage of blood vessels by astrocytic endfeet is reduced in major depressive disorder. Biol. Psychiatry.

[118] Lee T.S., Eid T., Mane S., Kim J.H., Spencer D.D., Ottersen O.P., de Lanerolle N.C. (2004). Aquaporin-4 is increased in the sclerotic hippocampus in human temporal lobe epilepsy. Acta Neuropathol..

[119] Binder D.K., Oshio K., Ma T., Verkman A.S., Manley G.T. (2004). Increased seizure threshold in mice lacking aquaporin-4 water channels. Neuroreport.

[120] Edmonson C., Ziats M.N., Rennert O.M. (2014). Altered glial marker expression in autistic post-mortem prefrontal cortex and cerebellum. Mol. Autism.

